# Leveraging artificial intelligence to reduce diagnostic errors in emergency medicine: Challenges, opportunities, and future directions

**DOI:** 10.1111/acem.15066

**Published:** 2024-12-15

**Authors:** R. Andrew Taylor, Rohit B. Sangal, Moira E. Smith, Adrian D. Haimovich, Adam Rodman, Mark S. Iscoe, Suresh K. Pavuluri, Christian Rose, Alexander T. Janke, Donald S. Wright, Vimig Socrates, Arwen Declan

**Affiliations:** ^1^ Department of Emergency Medicine Yale School of Medicine New Haven Connecticut USA; ^2^ Department of Biomedical Informatics and Data Science Yale University School of Medicine New Haven Connecticut USA; ^3^ Department of Biostatistics Yale School of Public Health New Haven Connecticut USA; ^4^ Department of Emergency Medicine University of Virginia Charlottesville Virginia USA; ^5^ Department of Emergency Medicine Beth Israel Deaconess Medical Center Boston Massachusetts USA; ^6^ Department of Medicine Beth Israel Deaconess Medical Center Boston Massachusetts USA; ^7^ Department of Emergency Medicine Stanford School of Medicine Palo Alto California USA; ^8^ Department of Emergency Medicine University of Michigan Ann Arbor Michigan USA; ^9^ Program in Computational Biology and Biomedical Informatics Yale University New Haven Connecticut USA; ^10^ Department of Emergency Medicine Prisma Health—Upstate Greenville South Carolina USA; ^11^ University of South Carolina School of Medicine Greenville South Carolina USA; ^12^ School of Health Research Clemson University Clemson South Carolina USA

## Abstract

Diagnostic errors in health care pose significant risks to patient safety and are disturbingly common. In the emergency department (ED), the chaotic and high‐pressure environment increases the likelihood of these errors, as emergency clinicians must make rapid decisions with limited information, often under cognitive overload. Artificial intelligence (AI) offers promising solutions to improve diagnostic errors in three key areas: information gathering, clinical decision support (CDS), and feedback through quality improvement. AI can streamline the information‐gathering process by automating data retrieval, reducing cognitive load, and providing clinicians with essential patient details quickly. AI‐driven CDS systems enhance diagnostic decision making by offering real‐time insights, reducing cognitive biases, and prioritizing differential diagnoses. Furthermore, AI‐powered feedback loops can facilitate continuous learning and refinement of diagnostic processes by providing targeted education and outcome feedback to clinicians. By integrating AI into these areas, the potential for reducing diagnostic errors and improving patient safety in the ED is substantial. However, successfully implementing AI in the ED is challenging and complex. Developing, validating, and implementing AI as a safe, human‐centered ED tool requires thoughtful design and meticulous attention to ethical and practical considerations. Clinicians and patients must be integrated as key stakeholders across these processes. Ultimately, AI should be seen as a tool that assists clinicians by supporting better, faster decisions and thus enhances patient outcomes.

## INTRODUCTION

Diagnostic errors present a formidable challenge in health care, as they contribute to significant patient safety risks, morbidity, mortality, and rising health care costs. The National Academies of Sciences, Engineering, and Medicine (NASEM) report, “Improving Diagnosis in Health Care,” highlights the urgent need for research and interventions to address these concerns, noting “It is likely that most people will experience at least one diagnostic error in their lifetime, sometimes with devastating consequences.”^1^

**FIGURE 1:**
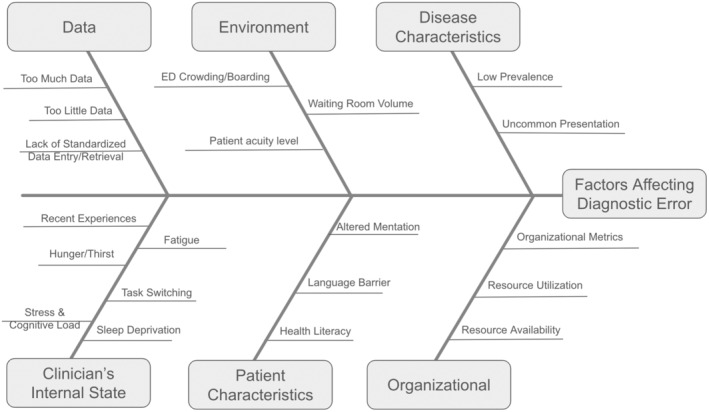
Factors that affect diagnostic error. Both internal and external factors increase diagnostic error in the ED setting. ECs’ internal states may be stressed by physiological challenges like hunger and fatigue, emotional impacts from prior experiences, and intrinsic skills like task switching. The external ED environment is replete with stimuli that can distract and strain clinicians, with ensuing risk of diagnostic error. Factors that appear to be intrinsic to the ED, like inadequate patient data, time and waiting room pressure, high decision frequency, and even noise can all increase the risk of diagnostic error. Similarly patient‐specific and disease‐specific factors make decision making more difficult while systemic organizational dysfunction and pressures can make diagnostic decision making into a hazardous exercise. EC, emergency clinician.

The emergency department (ED) is a uniquely demanding environment for accurate diagnosis (Figure 1). The heuristics of emergency clinicians (ECs) are continually challenged by high‐stakes decision making and strained by numerous external contextual factors. ECs make thousands of decisions daily, often under extreme time pressures and amid considerable variability in patient encounters.^2^, ^3^, ^4^, ^5^ The chaotic backdrop of the ED, replete with constant interruptions and distracting stimuli, exacerbates the difficulty of making accurate diagnostic decisions.^6^, ^7^ Moreover, clinicians must cope with circadian rhythm disruptions and the impact of prior high‐stakes experiences.^8^, ^9^, ^10^ These factors collectively contribute to a decision‐making landscape riddled with cognitive biases, variation, and fatigue.^11^ Attempts to decrease diagnostic error, for example, via education, debiasing, decision training, and decision support, have met with variable success.^7^, ^12^

In this paper, we discuss opportunities and challenges for artificial intelligence (AI) to enhance the accuracy of ECs’ diagnostic decision making via clinician‐oriented, patient safety–centric implementations. Thus far, AI tools have been difficult to translate into effective clinical use. We highlight the limited literature available on the use of AI for supporting ECs’ diagnostic decisions and describe the challenges that must be overcome to develop AI as a human‐centered tool that supports diagnostic decision making. Since AI should be developed as an integrated, embedded clinical tool that supports clinicians' cognitive strategies, we conceptualize AI support for diagnostic decisions within the dual‐process theory framework, where human thinking combines fast, intuitive, and automatic processing (System 1) with a slower, more deliberate, and analytical approach (System 2).^7^, ^13^, ^14^ We focus on three key sequential domains of decision making within the ED environment where AI could make a substantial impact:

1. *Improving available information to make a decision—*We explore how AI can support the information gathering processes of ECs by automating data gathering and summarizing relevant patient information.^15^, ^16^ Using AI to streamline information gathering can provide clinicians with quick access to pertinent patient data without extraneous details. This approach mitigates the cognitive overload common in the ED environment, reducing the analytic work demanded of System 2 and freeing cognitive resources for efficient System 1 processing.
2. *Supporting information synthesis with clinical decision support (CDS)—*After initial information gathering, AI can provide focused real‐time diagnostic support to complement the clinician's intuition with inductive analyses. By matching complex patterns and prioritizing differential diagnoses, AI can help mitigate cognitive biases to improve clinical decision accuracy, serving as a vital System 2 support in a predominantly System 1 environment.
3. *Facilitating education and feedback within quality improvement (QI)*—We examine how AI can facilitate rapid diagnostic outcome feedback and education through integration with QI initiatives. By integrating automated screening, trigger tools, and hierarchical screening within rapid QI feedback loops, AI can help refine ECs’ diagnostic acumen across System 1 and System 2, ultimately leading to improved patient outcomes and enhanced patient safety.

## DUAL‐PROCESS THEORY, EMERGENCY CARE, AND THE POTENTIAL BENEFITS OF AI

**FIGURE 2:**
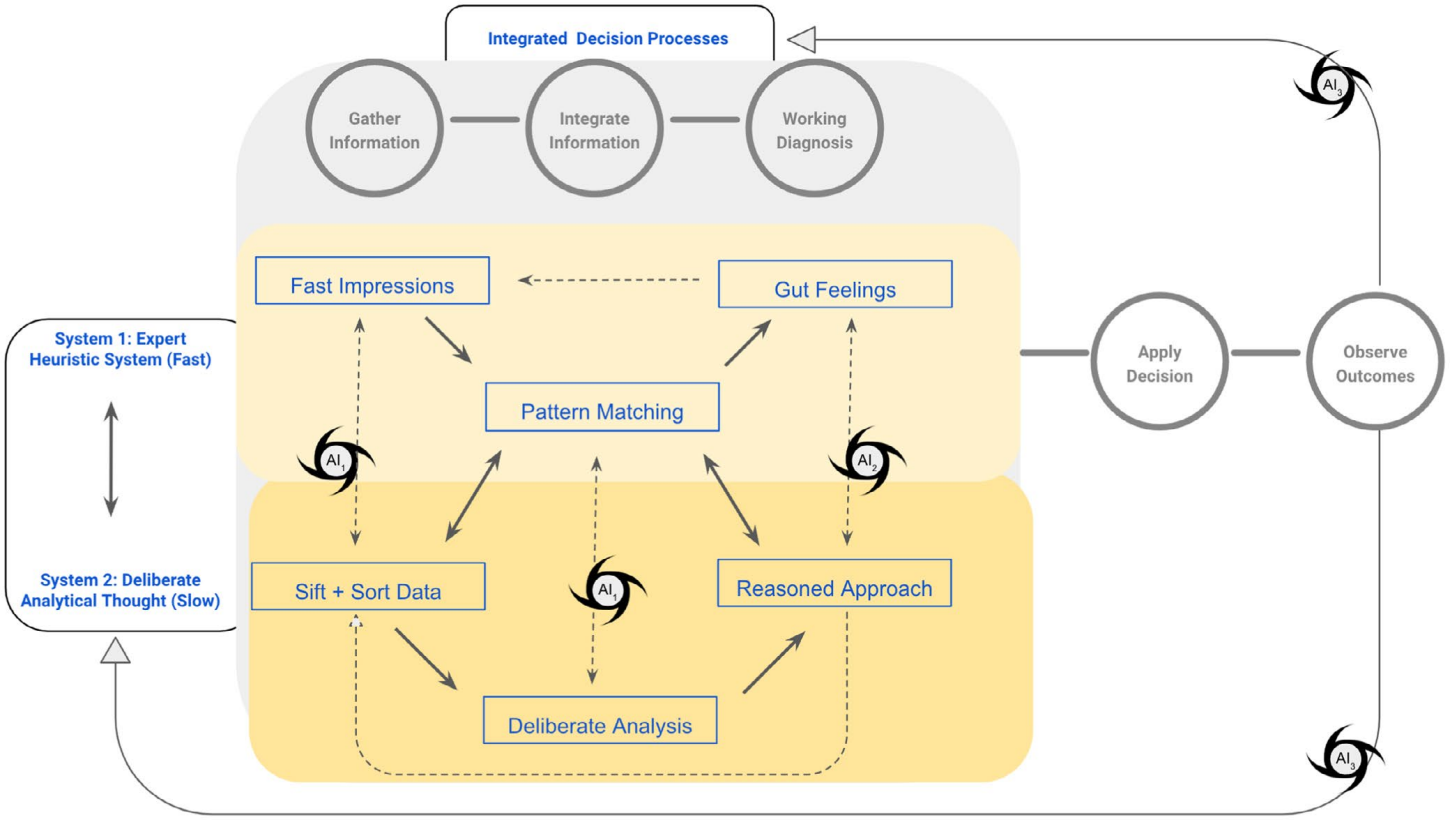
Integrated dual‐process decision model. Dual‐process theory proposes that two parallel cognitive systems contribute to decisions. System 1 describes rapid heuristic decision making that improves dramatically with expertise and uses pattern matching extensively. System 2 describes deliberative analytic thought that is much slower and that is thought to train and correct System 1 processes. While we now believe that the two systems cooperate, the two processes offer a helpful approach to optimizing AI‐based decision support tools. In this model, AI tools like NLP and LLMs could support information gathering and synthesis (AI_1_) by summarizing and collating information from across the EHR. AI‐driven tools that leverage LLMs, XAI, and RAG can support accurate working diagnoses (AI_2_) by providing probability‐weighted differential diagnosis lists and linking clinicians to relevant evidence. After decisions are applied and outcomes are observed, AI‐driven feedback loops (AI_3_) can help identify, filter, and analyze potential diagnostic errors to enhance quality improvement processes; the resulting outcome feedback and education can support future diagnostic accuracy by educating System 2 analyses and enhancing System 1 expertise. EHR, electronic health record; LLM, large language model; NLP, natural language processing; QI, quality improvement; RAG, retrieval‐augmented generation; XAI, explainable AI.

Dual‐process theory suggests that human decisions integrate two cognitive operations, or systems. System 1 provides rapid, intuitive judgments; its accuracy depends on expertise. System 2 offers resource‐intensive, data‐driven deliberations (Figure 2). Both systems are vulnerable to disruptions from individual, temporal, data‐related, and environmental factors.^17^, ^18^, ^19^ These systems appear to blend synergistically in the real‐world setting, especially within ED diagnostic workflows that intertwine direct patient evaluation (System 1), information review (System 2), and outcome feedback and cognitive review strategies (System 2) across various levels of intuitive expertise (System 1). While both systems are intrinsically error‐prone and contextually fragile, their integrated operation offers some protection from diagnostic decision errors. This synergy may reduce but does not entirely eliminate diagnostic errors. The pressures of the chaotic ED environment can destabilize diagnostic decision processes, since emergent patient presentations that require rapid, high‐stakes decisions are combined with incomplete information, frequent interruptions, and emotional strain that overload cognitive processes.^1^, ^4^, ^11^, ^12^, ^13^, ^14^, ^15^, ^16^, ^17^, ^18^, ^19^, ^20^, ^21^, ^22^, ^23^, ^24^, ^25^, ^26^, ^27^, ^28^, ^29^, ^30^, ^31^, ^32^, ^33^, ^34^, ^35^, ^36^, ^37^, ^38^, ^39^, ^40^, ^41^, ^42^, ^43^, ^44^, ^45^, ^46^, ^47^, ^48^, ^49^, ^50^, ^51^, ^52^, ^53^, ^54^ As a result, optimizing AI tools that can seamlessly support diagnostic decision making becomes crucial. By aligning AI with the natural cognitive processes of System 1 and System 2, AI tools can help enhance diagnostic accuracy and reduce errors, providing critical support in the demanding ED environment.

## IMPROVING AVAILABLE INFORMATION TO MAKE A DECISION

### Challenges in gathering clinical information

In the chaotic ED environment, clinicians must rapidly assimilate diverse data sources such as clinical history, laboratory results, and imaging results. To summarize relevant information for each patient, ECs must explore and sort fragmented information that may be spread across multiple platforms, often under extreme external pressures. These external pressures, such as fragmented data sources, time pressure, and frequent interruptions, contribute to cognitive overload, increasing the chance of diagnostic error and associated harm.^11^, ^17^ Other clinician‐specific factors, like fatigue, sleep debt, hunger, and affect, may also limit clinicians’ information gathering in the clinical environment.^11^ The lack of standardized data entry and information retrieval processes can also cause clinicians to overlook or underutilize critical information.^18^

The exact role of information retrieval via electronic health record (EHR) review in diagnostic safety remains incompletely understood. Since prior studies have not directly studied how EHR review contributes to diagnostic error,^19^, ^20^, ^21^ its impact may be confused with errors in clinical judgment^21^ or faulty information management.^19^ However, current evidence suggests that EHR review promotes high‐quality care for conditions like diabetes and hypertension.^22^ Furthermore, health information exchanges, which support EHR review across health systems, may reduce redundant medical workups,^23^, ^24^ improve care quality, and reduce costs in the ED setting.^25^, ^26^

Even though EHR review promotes high‐quality care, ED attending physicians devote only about 1 min per patient to chart review.^27^ National trends toward increasing ED patient acuity^28^ and comorbidity indices^29^ increase both time pressure and diagnostic complexity. Thus, rapid AI summaries of clinical charts may help clinicians to efficiently identify salient details from patients’ records.^30^

### Information summarization and synthesis

If developed as tools to summarize and collate patient information, AI‐driven systems could help ECs by providing accurate information summaries. Most existing textual extraction tools are optimized to summarize the biomedical literature,^31^ AI tools like large language models (LLMs) are currently being developed to summarize and collate patient information that is spread across the EHR.^32^ Natural language processing (NLP) tools can systematically extract and highlight pertinent details such as past diagnoses, treatment plans, and relevant patient history^33^ and thus could in theory reduce the likelihood of missing critical information. Though early data on LLM‐derived summarizations revealed significant variation in output,^30^ validated summarization tools could provide a coherent and concise overview of a patient's history, thus streamlining the hypothetico‐deductive processes of clinical decision making.

AI's role extends beyond simple summarization to the organized collation of diagnostic information. For instance, in patients presenting with chest pain, AI can seamlessly gather relevant data points, including recent troponin levels, echocardiograms, and catheterization reports.^34^ By automatically prioritizing the most recent and clinically relevant data, AI could ensure that clinicians are equipped with information essential for rapid decision making, while filtering out extraneous or redundant details. However, even carefully designed AI tools may not accurately detect essential information. They may fail to accurately sort or prioritize information or may be unable to manage chronological relevance.^30^ As this challenge is addressed, AI tool design could leverage AI's ability to generate dynamic and customizable information displays that can enhance clinical context awareness by adapting displayed information to the patient's acuity and by tailoring the information to the clinical context.^35^ By grouping data by pathophysiological categories and integrating medications, lab results, and imaging studies, AI can reduce the cognitive burden associated with synthesizing disparate information, thus facilitating quicker, more accurate decision making. AI can prioritize and flag critical data, such as abnormal lab results or signs of patient deterioration, to draw attention to urgent information. For example, context‐aware dashboards can highlight relevant data trends, such as the progression of lab values, enhancing the clinician's ability to track patient status over time.^36^ These strategies have been applied in machine learning AI sepsis detection tools such as SepsisWatch and TREWS, which improve sepsis detection, treatment timing, and outcomes.^37^, ^38^, ^39^ To successfully expand beyond local health care systems, these tools would need to be validated across populations and implemented across institutional data sets. These hurdles require addressing core “Big Data” challenges such as variability in data structures or ontologies. Some of these practical challenges might be overcome with vendor collaborations that leverage shared EHR data structures, as illustrated by the Epic Sepsis Model that is now integrated into the Epic EHR. However, this tool has met with variable success, and vendor‐specific models may not adequately address the entire population.^40^, ^41^, ^42^ These AI tools must also optimize accurate, thorough, and concise summarization without risking hallucination.^32^

This strategy can reduce the need for manual data retrieval and alerts clinicians to potential gaps or inconsistencies in the available information. In addition, AI can cross‐reference data across multiple EHR sources, such as radiology systems and laboratory databases, to detect patterns or correlations that may not be immediately apparent, prompting further investigation and minimizing the risk of missed diagnoses.

## ACTING AS DIAGNOSTIC ASSISTANCE THROUGH FOCUSED CDS

AI‐enabled CDS systems focused on particular decisions points that strongly leverage System 2 have the potential to lessen the cognitive load felt by ECs, reduce medical errors, increase patient throughput, and improve quality of care.^43^, ^44^, ^45^ However, CDS design and implementation require careful consideration to ensure that the “right” information is presented in the “right” format at the “right” time.^46^, ^47^ AI‐based CDS tools also should be audited and adapted using AI fairness toolkits to ensure that they do not induce unjust discrimination.^48^, ^49^

Prior work shows that clinicians are quite accurate when their initial impressions include the correct diagnosis.^13^ We posit that by expanding and prioritizing initial diagnostic differentials, AI‐driven CDS can expand ECs’ initial diagnostic impressions and thus improve diagnostic accuracy. By expanding differential diagnoses, CDS could support rapid System 1 pattern matching while activating System 2 analytic and feedback processes. AI‐driven CDS tools can automatically generate a list of differential diagnoses based on the patient's presenting symptoms and clinical history. LLMs can produce a more complete differential diagnosis faster than clinicians in the experimental setting.^50^, ^51^ AI‐based CDS differentials could mitigate common cognitive biases, such as anchoring bias or availability bias, by suggesting alternative diagnoses and encouraging clinicians to consider a broader range of possibilities. Even though existing evidence is encouraging, AI CDS differentials are prone to inherit societal biases from their training data. They therefore must be carefully and continuously evaluated to ensure that they do not exacerbate existing health inequities.^52^, ^53^, ^54^

In addition to broadening the differential diagnosis, AI‐based CDS systems can provide additional context for decision making through uncertainty estimation and analysis explanation. Proper calibration of AI models before implementation can significantly improve their effectiveness by providing probability scores for various diagnoses and helping ECs gauge the likelihood of different conditions.^55^ Generalized transformers such as LLMs seem to be well calibrated after initial training and may thus be able to provide effective recommendations under uncertainty without requiring additional training.^56^, ^57^ By presenting a range of differential diagnoses along with associated probabilities, AI can inform decision making in uncertain or ambiguous cases. Such uncertainty quantification can indeed improve trust calibration in human + AI systems; there is some evidence that model‐estimated probabilities are not enough.^58^ In such cases, explainable AI (XAI) methods can enable improved trust in AI‐enabled CDS tools. When developed with stakeholder involvement, CDS tools with XAI improve trust in AI systems and potentially improve patient care.^59^, ^60^, ^61^, ^62^ With the advent of text‐based AI systems such as LLMs, XAI methods have become more digestible to care providers in the ED.^63^

A major advantage to AI‐driven CDS tools in diagnostics is their ability to summarize large amounts of information and provide recommendations. In the ED, this may include integration with evidence‐based clinical practice guidelines, for example, via machine learning–based triage risk stratification for patients with chest pain.^64^ Since language models have a strong capacity for clinical summarization, these information summarization pipelines can be integrated directly with guidelines or other forms of medical evidence in a retrieval‐augmented generation (RAG) framework.^65^, ^66^, ^67^ In this context, a RAG organizes and extracts relevant data from a larger data source, such as a guideline index or a medical textbook. Thus, the language model can present relevant evidence‐based information and guideline summaries to the EC at opportune moments, such as before a major decision. In addition to synthesizing and presenting information to an EC at the “right” time, AI‐enabled CDS can enhance diagnostic consistency by providing standardized diagnostic criteria and suggestions across all providers, reducing variability across different clinicians or shifts.^68^, ^69^ This consistency is particularly important in the ED, where multiple providers may be involved in the care of the same patient over time. AI‐driven CDS tools can facilitate collaboration between specialists by providing a shared platform for diagnostic suggestions and decision support, ensuring that all team members are aligned in their diagnostic approach.^70^

These early directions offer hope for improving diagnostic accuracy. AI‐driven diagnostic CDS tools could support ECs’ System 1 processes by parsing and condensing large amounts of information to enable more informed intuitive decision making while avoiding cognitive biases. They can also support System 2 thinking by providing guideline‐grounded information, uncertainty estimations, and explanations that allow for more deliberate and analytical EC–AI collaboration and decision making. We emphasize the importance of stringent development, testing, and postimplementation quality tracking. AI tools do not yet fall under clear regulatory oversight and suffer many risks of failure, ranging from variable output due to probabilistic mechanisms; prompt variability; sycophancy; inability to define, prioritize, and highlight essential information; and “glitches” similar to AI hallucinations.^32^ Diagnostic AI tools must be explored and validated thoroughly lest inferior algorithms compromise diagnostic accuracy and patient safety.

## FACILITATING EDUCATION AND FEEDBACK WITHIN QI

AI can enhance the System 2 feedback loops that develop System 1 intuition via rapid, intensive QI feedback. Analytic QI case review strengthens System 2 analyses and enhances System 1 experience‐based intuition. QI mechanisms that combine outcome feedback with relevant education improve diagnostic performance.^71^ AI tools can speed the feedback process by screening for quality gaps, exploring clinicians’ EHR practice patterns, and reflexing relevant summaries to clinicians. Thus, embedding AI‐driven feedback loops into ED QI frameworks can enhance systematic and personal diagnostic accuracy.

AI‐driven feedback loops may be particularly helpful to address the challenges inherent in ED QI efforts. ED QI infrastructure often combines outcome feedback with education;^72^, ^73^ existing tools support best practices for managing pneumonia and sepsis,^74^ timely treatment of acute myocardial infarction,^75^ general throughput,^76^ documentation quality,^77^ adverse events,^78^ and a variety of other targets.^79^ Recent work suggests that an LLM can retrospectively abstract and categorize sepsis for QI review, though the AI‐associated hallucinations noted within the small pilot sample suggest that this promising development will require further refinement.^80^

QI interventions may also include targeted feedback for individual practicing clinicians. These feedback mechanisms allow clinicians to learn from past cases to improve future performance. However, clinical reasoning feedback in emergency medicine (EM) is uniquely challenging.^81^ Best practices in QI feedback loops may be difficult to implement because variable ED schedules preclude timely, frequent feedback; heterogeneous patient presentations require many targets for action rather than limited clear targets; and attention gravitates easily to unexpected morbidity rather than best practices.^72^ Feedback is often limited to departmental morbidity and mortality conferences, which can seem punitive and may prime participants to practice defensively.^82^ Individualized feedback and education require significant resources and are thus difficult to implement at scale.^83^ The efficiency and scalability of AI may mitigate these barriers to ED QI feedback and education.

### Automated case screening for diagnostic error

**FIGURE 3:**
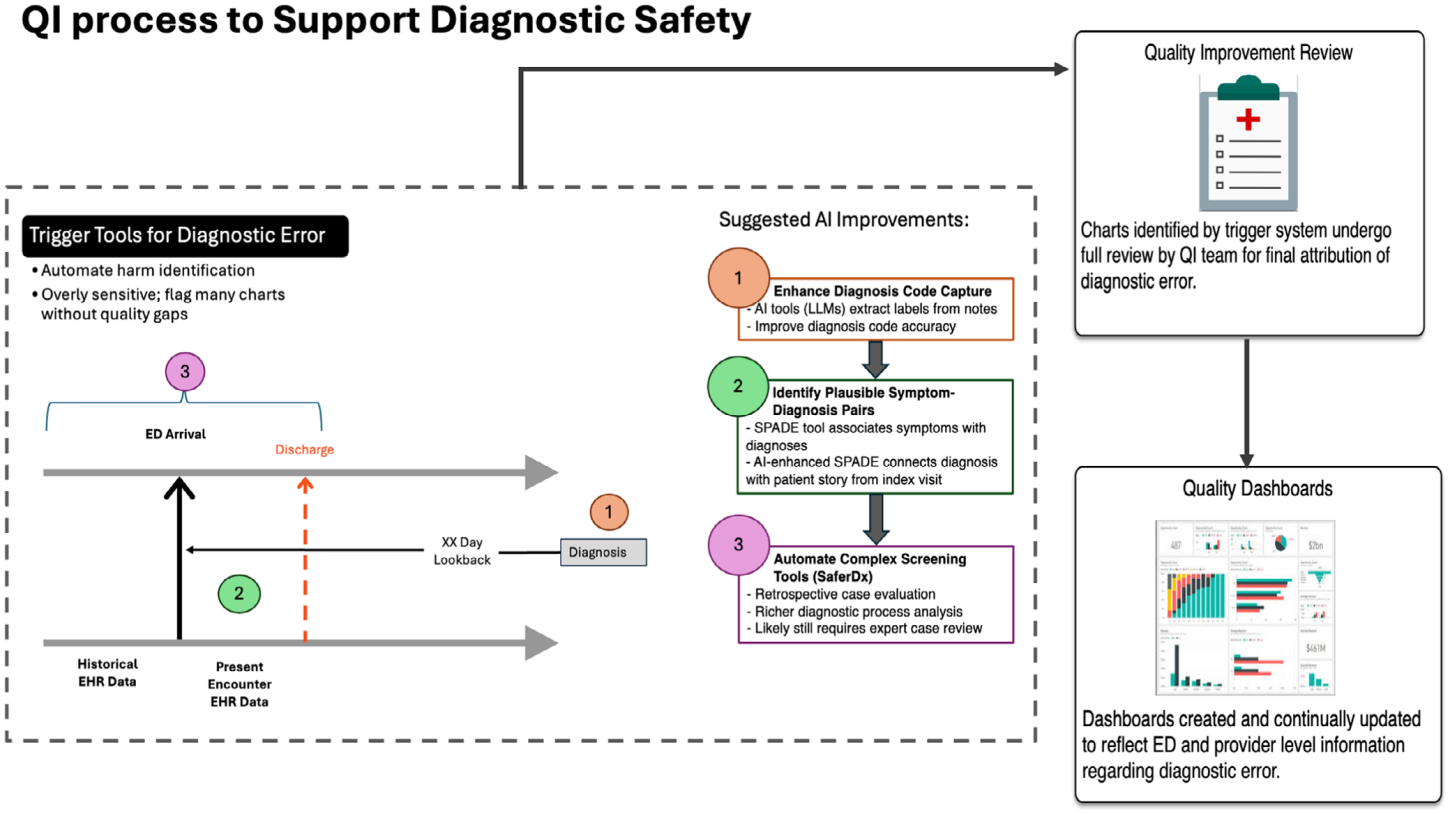
Hierarchical AI‐powered “trigger tools” can scale QI analyses. Serial implementation of AI tools in a hierarchical screening process could support efficient, scalable QI processes. Consideration of a case for QI evaluation could arise from human referrals or AI screening algorithms. Since these methods are overly sensitive, and often identify charts that do not contain quality gaps, this initial screen can be filtered through three tiers. First, LLM tools can extract diagnostic labels from text and other data to accurately characterize outcomes as diagnosis codes. Then, cases can be screened for plausibility based on symptom‐disease matching using the SPADE tool. This step would eliminate cases where patients presented repeatedly but where symptoms reported at prior visits were not plausibly linked with the outcome of concern. Finally, AI tools could be used to complete the SaferDx case evaluation instrument. This step would prepare cases for efficient and thorough human QI review. Cases that prompt outcomes feedback could then be funneled into AI‐driven feedback loops to drive education to individual physicians. AI, artificial intelligence; LLM, large language model; QI, quality improvement.

Recently developed AI trigger tools, which were designed to tailor case lists and measure harm in the ED setting, may ease the burden of individualized feedback and improve scalability.^84^ While these tools do help by automating harm identification, they are overly sensitive and flag many charts that do not contain quality gaps. These screening processes will need to be improved for efficient implementation. Here, we describe a solution using hierarchical screening processes for AI‐assisted diagnostic error detection (Figure 3).

The first hierarchical level is to enhance diagnosis code capture. Diagnosis labels (e.g., International Classification of Diseases codes) are often incomplete or inaccurate, but identifying and labeling a clinician's diagnostic impression is essential for measuring diagnostic quality. AI tools such as LLMs can effectively extract labels from free text sources such as radiology notes to improve diagnosis code accuracy for downstream applications.^85^

The next hierarchical level is to identify plausible pairs of symptoms and diagnoses. For example, in a case of delayed diagnosis of pulmonary embolism (PE), a prior visit involving chest pain could plausibly be related to the diagnosis of PE and could thus be identified as a relevant presentation, whereas a prior presentation with wrist pain would be excluded. The Symptom‐Disease Pair Analysis for Diagnostic Error (SPADE) tool identifies diagnostic error by associating symptoms identified at one health care encounter and diagnoses identified at subsequent encounters.^86^ At its simplest, SPADE can be used to relate diagnostic codes. We envision future development so AI‐enhanced SPADE can connect the eventual diagnosis with a complete patient history as summarized by a language model.

The third and most sophisticated hierarchical level is to automate more complex screening tools like SaferDX. SaferDx is a structured data collection instrument that evaluates cases retrospectively for opportunities to make correct and timely diagnoses.^87^ This tool has been refined and extended across specific clinical conditions^88^ and in implementation as part of learning laboratories.^89^ In contrast to trigger tools, which rely on automated review of EHR data, SaferDx provides a much richer evaluation of the diagnostic process. This process requires significant resource investment for individualized expert case review.

## CHALLENGES AND LIMITATIONS

While AI might decrease diagnostic errors by supporting information gathering, information integration, and feedback loops, realizing this potential is challenging. Barriers to successful AI tools reflect tool scarcity, inadequate infrastructure, complex stakeholder networks, design and implementation decisions, intrinsic human limitations, and technological limitations of AI systems (Table 1).

**TABLE 1:** Challenges and future directions.

Challenge	Potential risks	Proposed solution	Research gaps	Stakeholder engagement strategies
AI trust/distrust	Overreliance on AI or rejection of AI outputs	Trust calibration mechanisms, human–AI collaboration models	How to optimally balance trust in AI vs. human decision making	Continuous clinician education, codevelopment of AI tools with health care providers
Cognitive overload from AI	Cognitive fatigue, impaired clinical judgment	Streamlined AI interfaces, decision‐support prioritization	Impact of interface design on clinician cognitive load	Involve clinicians in interface design, iterative feedback loops during development
Regulatory gaps	Lack of oversight leading to unsafe implementations	Development of AI regulatory standards and guidelines	How to create adaptive regulatory frameworks for AI in health care	Advocacy and policy reform engagement with regulators, collaboration with legal experts
Explainability	Clinicians not understanding AI outputs, leading to low adoption	XAI models with clear decision rationale	Understanding the extent to which XAI improves trust and accuracy	Collaborate with ethics and communication experts for effective XAI implementation
Bias in AI models	Reinforcing health disparities, unethical AI recommendations	AI fairness audits, algorithmic transparency	Effective strategies to mitigate bias in real‐world data sets	Regular bias audits, partnerships with ethics researchers, community feedback loops
Data integration	Fragmented data across systems, incomplete data sets	Interoperability frameworks, HIE	Best practices for cross‐institutional data sharing and harmonization	Early collaboration with hospital IT departments and policymakers for HIE standardization
Cognitive bias mitigation	AI reinforcing clinician biases (e.g., anchoring, confirmation bias)	AI systems designed to counteract human cognitive biases	Determining optimal ways for AI to challenge clinician biases	Close collaboration with cognitive psychologists and clinicians to design bias‐mitigating features
Privacy concerns	Data breaches, patient mistrust in data usage	Strong encryption, patient‐centered consent frameworks	How to align AI data practices with evolving privacy laws (e.g., GDPR, HIPAA)	Engage with legal and data privacy experts early, public transparency initiatives
Workflow integration	Disruption of clinical workflows, reduced efficiency	User‐centered design of AI tools, phased AI implementation	Understanding the optimal points for AI integration in emergency settings	Regular clinical workflow evaluations, phased rollouts with feedback loops from ECs
Training and education	Low clinician proficiency in using AI tools	Comprehensive AI training programs, continuous learning	Best pedagogical methods for teaching AI tool usage in health care	Collaborative training programs with medical education experts, incorporating AI into ongoing professional development

Abbreviations: ECs, emergency clinician; HIE, health information exchange; XAI, explainable AI.

AI implementations across EM—and indeed, broadly across health care—are limited by the scarcity of widely disseminated and effective tools. The sepsis predictions systems described above demonstrate the value of AI tools across individual health care systems, but the Epic Sepsis Model has been widely criticized for variable performance and lack of transparency in real‐world environments.^38^, ^39^, ^40^, ^41^ While AI tools are being developed and tested locally, few AI tools have achieved both broad dissemination and consistent clinical impact.

Widespread AI adoption is also hindered by inadequate infrastructure for AI development, implementation, and monitoring. Most health care systems lack the machine learning operations (MLOps) infrastructure necessary to deploy and maintain AI tools effectively (e.g., SepsisWatch implementation^38^). High‐quality data sets, real‐time data pipelines, and skilled data scientists are necessary to develop, implement, and maintain AI tools, yet few institutions have enough of these resources. Widely inadequate infrastructure creates significant gaps between research and real‐world application. Moreover, health care institutions are often culturally unable to integrate AI systems seamlessly within live clinical workflows. For example, cultural prohibitions and systemic barriers often prevent researchers from accessing real‐time systems. Since researchers are often leaders in evidence‐based innovation and critical evaluation of AI tools, these barriers effectively separate the development and validation of AI tools from operational implementation. The lack of critical systemic and cultural infrastructure development limits health care systems’ ability to iterate, validate, and monitor AI systems. Although other organizations across the healthtech marketplace may be able to support the needed infrastructure, each organization must also address existing systemic and cultural barriers. Furthermore, since the real‐time data needed for AI implementations are collected within vendor‐specific closed box systems, organizations must negotiate restrictive marketplace silos and operability challenges. Thus, the interface between healthtech and ED health care must be further developed to optimize AI tool development. Unless these barriers are addressed during transitions to learning health systems, AI tools may remain difficult to test, implement, and scale.

Implementations must also consider optimal design strategies centered around clinician workflows. Although clinicians’ workflow patterns often reflect environmental complexity, information gathering via EHR review typically occurs early in clinician workflows.^90^ Interruptions, including AI alerts, fragment physician workflows, trigger workarounds, decrease clinician efficiency, and lead to clinical errors.^11^, ^91^ Thus, particularly in high‐pressure environments like the ED, effective integration of AI into existing diagnostic processes requires careful consideration to ensure that AI tools complement rather than disrupt the clinician's workflow.

Similarly, AI tools such as CDS systems are meant to assist, not confuse, clinicians’ diagnostic decision‐making strategies.^90^, ^91^ Prior CDS research has often neglected important factors such as usability and workflow integration, including core competencies such as the “five rights” (right information, person, format, channel, time).^47^, ^92^ Poorly implemented CDS systems have often led to frustration.^93^ AI outputs must be optimized for EC workflows and needs.^93^ Since AI tools like LLMs produce output that varies probabilistically and in response to prompt inputs,^94^ outputs must also be optimized to communicate accuracy and confidence estimates that reflect this intrinsic variability.

Integrating this probabilistic metadata may be challenging for ECs, especially under the cognitive overload and decision fatigue that commonly impede interpretation and translation of new information in the chaotic ED setting.^95^ These factors increase diagnostic errors and adverse patient events.^11^, ^17^, ^96^, ^97^ Poor AI implementation can exacerbate this problem, whether via complex visualizations or frequent interruptions.^17^, ^91^ Further, ECs often use heuristics rather than strict numerical or descriptive probability outputs, and they distort probabilities at numerical extremes.^98^, ^99^ Well‐designed, validated visual information displays can help communicate statistical information, but clinicians’ interpretation of probability is fragile and depends on the type of illustration provided.^100^, ^101^, ^102^ AI output strategies will need to be carefully developed and tested to provide usable information in appropriate formats that offload, rather than overload, ECs.^15^

Even though AI, particularly ML, is considered helpful for improving the diagnosis of rare diseases, AI differential diagnosis generators are still regrettably inaccurate for unusual and challenging diagnoses.^103^, ^104^ Rare disease detection requires extensive, high‐quality training data that may not be available for rare diseases, so AI may have “blind spots” that limit recognition of atypical presentations or rare conditions.^105^

Integrating AI into clinical practice also introduces risk across outcome and feedback loops. Clinicians can trust or distrust AI output excessively. Excessive trust risks decreasing alertness for subtle clinical clues, introducing anchoring bias around AI differentials, or failing to recognize inconsistencies. For example, clinicians may struggle to proofread and correct inconsistencies in ambient AI scribe output.^106^ Conversely, excessive distrust results in poor adoption and limits effectiveness. Skepticism may arise from concerns about AI accuracy or fear of technology supplanting human judgment.^107^ Clinicians may ignore AI‐suggested differential diagnoses due to concerns about LLM hallucinations^108^ or well‐founded suspicions that AI may perpetuate biases from training data sets;^93^ they may also decline to implement new technologies due to negative reviews by colleagues.^109^, ^110^

## FUTURE DIRECTIONS FOR IMPLEMENTATION AND RESEARCH

Given these challenges, optimizing AI support for diagnostic processes requires careful, strategic implementation and research. Health care systems must apply collaborative, data‐driven learning health system processes to test and scale AI tools wisely. Health care systems must also develop infrastructure wisely to optimize patient impact and maintain fiscal responsibility. EHR vendors must also balance useability against profit; EHR‐wide tools must be carefully validated before implementation. EHR and service vendors may also have the opportunity to support critical infrastructure across segments of the health care marketplace. To avoid increasing cognitive burden and introducing new cognitive errors, seamless workflow integration must be a priority. Future efforts should focus on user‐centered design approaches that align AI tools with existing clinical workflows, involving phased implementation with continuous feedback loops and customization options for different clinical environments.^111^ Developing optimal models for clinician–AI collaboration is crucial to ensure that these tools enhance rather than hinder clinical judgment. This necessitates comprehensive training programs for clinicians and strategies to address resistance to change.^112^

Finally, it will be crucial to objectively evaluate AI's impact on diagnostic accuracy in emergency medicine. Standardized metrics like diagnostic accuracy, time to diagnosis, patient outcomes, and clinician cognitive load are essential to assess AI's effectiveness in clinical practice. Longitudinal studies across diverse clinical settings should examine AI's effects on high‐risk or frequently misdiagnosed conditions. AI tool development must optimize diagnostic processes, probability communication, resource allocation, and ethical impact^104^ in parallel with stakeholder engagement and user education initiatives to improve patient care in the high‐pressure ED environment.

## CONCLUSIONS

Artificial intelligence offers a powerful tool for data‐intensive inductive, analytical information management.^113^ While emergency clinician decision accuracy is challenged by typical human biases as well as personal and environmental stressors, artificial intelligence can mitigate these stressors to support more robust clinical decision making and decrease diagnostic errors.^15^, ^114^ To be effective, artificial intelligence must be integrated within the context of emergency clinician decision processes and workflows. By streamlining information gathering, providing clinical decision support, and supporting feedback loops, artificial intelligence implementations can decrease emergency clinician cognitive overload, enhance evidence‐based diagnoses, and support emergency clinician quality improvement feedback loops. We recognize that, even in the context of an idealized learning health system, diagnostic errors are not perfectly preventable in the chaotic ED setting. Information limitations and natural disease progression will always limit diagnostic forecasting. Furthermore, experienced emergency clinicians know that their job is to provide stabilizing care, rule out immediate life threats, and help patients access care. The goal of artificial intelligence integration should be to support clinicians in making the best possible decisions with available information while continuously improving the diagnostic process. Ultimately, these targeted applications will enhance patient care and increase diagnostic accuracy. The future of diagnosis in emergency medicine must be collaborative, with artificial intelligence serving as an essential component of the diagnostic team. As we move forward, continuous education, feedback, and engagement with all stakeholders will be crucial to ensuring that artificial intelligence tools are effectively and ethically integrated into clinical practice.

## AUTHOR CONTRIBUTIONS

R. Andrew Taylor, Arwen Declan, and Rohit B. Sangal conceived the conceptual approach to the manuscript. All authors drafted the manuscript, and all authors contributed substantially to its revision. R. Andrew Taylor takes responsibility for the paper as a whole.

## FUNDING INFORMATION

RAT receives grant support from Beckman Coulter, Inc., for AI development and evaluation.

## CONFLICT OF INTEREST STATEMENT

The authors declare no conflicts of interest.

## Data Availability

Data sharing is not applicable to this article as no new data were created or analyzed in this study.
